# Perioperative management of infant inguinal hernia surgery; a review of the recent literature

**DOI:** 10.1111/pan.14726

**Published:** 2023-07-14

**Authors:** Fiona Taverner, Prakash Krishnan, Robert Baird, Britta S. von Ungern‐Sternberg

**Affiliations:** ^1^ College of Medicine and Public Health Flinders University Adelaide South Australia Australia; ^2^ Department of Anaesthesia and Pain Management Flinders Medical Centre Adelaide South Australia Australia; ^3^ Department of Anesthesia BC Children's Hospital Vancouver British Columbia Canada; ^4^ Department of Anesthesiology, Pharmacology and Therapeutics UBC Vancouver British Columbia Canada; ^5^ Division of Pediatric Surgery BC Children's Hospital Vancouver British Columbia Canada; ^6^ Department of Anaesthesia and Pain Medicine Perth Children's Hospital Nedlands Western Australia Australia; ^7^ Division of Emergency Medicine, Anaesthesia and Pain Medicine The University of Western Australia Perth Western Australia Australia; ^8^ Perioperative Medicine Team, Perioperative Care Program Telethon Kids Institute Nedlands Western Australia Australia

**Keywords:** anesthesia, inguinal hernia, pediatric, perioperative, surgery

## Abstract

Inguinal hernia surgery is one of the most common electively performed surgeries in infants. The common nature of inguinal hernia combined with the high‐risk population involving a predominance of preterm infants makes this a particular area of interest for those concerned with their perioperative care. Despite a large volume of literature in the area of infant inguinal hernia surgery, there remains much debate amongst anesthetists, surgeons and neonatologists regarding the optimal perioperative management of these patients. The questions asked by clinicians include; when should the surgery occur, how should the surgery be performed (open or laparoscopic), how should the anesthesia be conducted, including regional versus general anesthesia and airway devices used, and what impact does anesthesia choice have on the developing brain? There is a paucity of evidence in the literature on the concerns, priorities or goals of the parents or caregivers but clearly their opinions do and should matter. In this article we review the current clinical surgical and anesthesia practice and evidence for infants undergoing inguinal hernia surgery to help clinicians answer these questions.

## 1 INTRODUCTION

Inguinal hernia surgery is one of the most common electively performed surgeries in infants.^1^ An indirect inguinal hernia, such as occurs in children, is defined as a protrusion of abdominal contents (e.g. intestines, ovary, omentum) through a patent processus vaginalis. It occurs in 3%–5% of babies born at term and up to 15% of babies born before 33 weeks gestational age.^2^ The most common risk factors for development of an infant inguinal hernia include preterm birth (9%–20% risk), male sex (87% under the age of 1 year are male), very low birth weight, and mechanical ventilation.^3^, ^4^, ^5^

Even in asymptomatic infants, the presence of an inguinal hernia necessitates surgical repair due to the high risk of incarceration; in children under 1 year of age this has been reported at around 8%^3^ and the risk is highest in preterm children at around 20%.^5^, ^6^ Development of a hernia within 2 months of age significantly increases likelihood of developing a subsequent contralateral hernia following surgery,^7^ therefore bilateral repair is most commonly performed in preterm infants.

Neonates and infants have higher perioperative risks than older children due to differences in developmental physiology, as well as the increased likelihood of comorbidities associated with congenital abnormalities and preterm birth.^8^, ^9^ A review of perioperative mortality in an Australian tertiary pediatric centre showed that neonates (aged <30 days) have the highest risk of pediatric perioperative mortality of 180.1 per 10 000 anesthetics, compared with 40 deaths per 10 000 anesthetics in infants (31 days to <1 year) and 16 deaths per 10 000 anesthetics in older children 10–17 years.^10^ This was echoed in a similar, more recent study in another Australian centre showing perioperative mortality in neonates to be 227 per 10 000 anesthetics.^11^ Neonates and infants have a high rate of severe respiratory and cardiovascular adverse events under anesthesia of 35%, which is higher than older children.^1^, ^12^

Children born preterm account for around 10% of births worldwide. They have a higher rate of associated comorbidities such as chronic lung disease, retinopathy of prematurity, necrotising enterocolitis and neurologic conditions such as intraventricular hemorrhage and periventricular leukomalacia.^13^ Consequently, they have a greater likelihood of requiring surgery as well as having greater perioperative risks than infants born at term.

The common nature of inguinal hernia, combined with the high‐risk population involving a predominance of preterm infants makes this a particular area of interest for those concerned with their perioperative care. Despite a large volume of literature in the area of infant inguinal hernia surgery, there remains much debate amongst anesthetists, surgeons and neonatologists regarding the optimal perioperative management of these patients. The questions asked by clinicians include; when should the surgery occur, how should the surgery be performed (open or laparoscopic), how should the anesthesia be conducted, including regional versus general anesthesia (GA) and airway devices used, and what impact does anesthesia choice have on the developing brain? There is a paucity of evidence in the literature on the concerns, priorities or goals of the parents or caregivers but clearly their opinions do and should matter.

## 2 TIMING

Whilst the need for repair of inguinal hernia appears to reach universal agreement amongst the surgical community due to the risk of incarceration and resultant morbidity, there remains much doubt as to the ideal timing of the operation in infants with non‐incarcerated inguinal hernias. The decision regarding timing of repair is a balance of the surgical risk of incarceration favoring early repair against the perioperative anesthetic risk which favors delayed repair, as there is good evidence that the overall anesthetic and post operative respiratory risks are inversely related to post menstrual age.^1^, ^14^ A longitudinal population‐based study showed an overall incarceration rate of around 8% in infants, which was double the overall rate for children 0–15 years of age at 4%. This study did not find any difference in incarceration rates in preterm infants compared to those infants born at term, however it was not designed specifically to explore timing of surgery.^3^ In contrast to this, several studies, including a recent meta‐analysis of early versus delayed repair in preterm infants, showed a significant reduction in risk of incarceration with early repair, but a higher risk of post operative respiratory complications.^5^, ^15^ A large multicentre randomized trial comparing outcomes in preterm infants undergoing inguinal hernia surgery either prior to discharge or at 55–60 weeks post menstrual age aims to address this important question definitively and is nearing completion.^16^

The European Pediatric Surgeons' Association Evidence and Guideline Committee recently released a recommendation that postponing repair in infants until after discharge may be beneficial in preventing respiratory complications and hernia recurrence.^17^ However, a consideration must also be made of the social factors of the infants' family; where loss to follow up, significant financial hardship and long distance from their treating hospital, such as families living in regional and remote Australia or Canada, may necessitate repair prior to discharge to balance the overall best interests of the infant and their family.

## 3 SURGICAL TECHNIQUES LAPAROSCOPIC VERSUS OPEN

Traditionally, inguinal hernia repair in children and infants is performed with an open technique via the inguinal skin crease. While multiple eponymous variations exist with this approach, the fundamental principle is separating, ligating and ultimately removing the hernia sac as proximal as possible to the internal inguinal ring. This operation remains the most commonly performed elective procedure in pediatric general surgery; with as many as 20 million inguinal hernia procedures performed annually.^18^ The goal of the procedure is to permanently address the inguinal communication while minimizing intraoperative complications such as spermatic cord/vessel/ovarian injury or bleeding as well as postoperative complications such as haematoma, hydrocele, wound infection, and hernia recurrence. The technique is extremely reliable with very low morbidity rates.

With the advent of minimal access techniques, surgeons have employed multiple iterative changes to the technique of inguinal hernia repair. An early variation that has failed to gain large scale traction is to cannulate the hernia sac through the inguinal approach with a trocar followed by an angled laparoscope in order to inspect the contralatereal groin.^19^ This technique does not meaningfully alter the principles of addressing the ipsilateral groin via the traditional operative technique. It is designed to interrogate the contralateral internal ring for the presence/absence of a patent processus vaginalis—essentially an abnormal extension of peritoneal lining that constitutes a hernia sac. The presence of a patent processus vaginalis positively predicts the future development of a later metachronous hernia, although the exact risk of later hernia development remains undefined. A positive identification of a contralateral patent processus vaginalis during this hybrid laparoscopic procedure would typically trigger a groin exploration and open hernia repair for a heretofore “silent” hernia.

Increasingly, pediatric surgeons are choosing to both identify and repair pediatric inguinal hernias via conventional laparoscopic techniques with the camera port in the umbilicus. This allows for addressing the ipsilateral, presenting hernia without an open groin incision as well as inspection, and if required, repair of the contralateral side. Data from Europe and North America confirms that the laparoscopic approach is gaining in popularity^20^, ^21^, ^22^ although a true tipping point has not been reached and the open approach remains more popular. With the increasing advent of laparoscopic inguinal hernia repairs in children, a host of different technical variations have been described—an excellent compendium recently compiled by the Society of American Gastrointestinal and Endoscopic Surgeons lists 20 unique procedures.^23^ Importantly, these all adhere to the original surgical dictum of controlling the “neck” of the hernia at the internal inguinal ring and none of the repairs use mesh or other exogenous material to reinforce the closure.

Because of the plethora of surgical techniques, comparative data of outcome and complication rates are difficult to analyze. Indeed, multiple overlapping systematic reviews have selectively included or excluded different source data on the topic. Despite these variations, what is relatively clear is that the laparoscopic approach to addressing a pediatric inguinal hernia results in a comparable risk profile with regards to the ipsilateral presenting pathology while reducing the risk of a later metachronous hernia on the contralateral side.^24^, ^25^, ^26^ There may be differences in resource utilization rates when comparing the open and laparoscopic techniques: several studies suggest that a unilateral hernia may be more expeditiously addressed with the open approach whereas a bilateral hernia (or ipsilateral hernia with a contralateral patent processus vaginalis) can be repaired more quickly via laparoscopy.^27^, ^28^ Furthermore, laparoscopy may be advantageous in addressing instances of incarcerated inguinal hernias by allowing for easier reduction and inspection of incarcerated contents; a shorter length of stay and less overall complications has been documented in a recent systematic review.^29^

## 4 ANESTHETIC TECHNIQUES

Many different combinations of anesthesia techniques have been tried and reported for infant inguinal hernia repair, ranging from a spinal block or caudal block alone or with sedation, to GA with or without a caudal or other regional block. With GA, airway management varies widely from use of a face mask to a laryngeal mask airway or endotracheal tube. The large variation in clinical practice highlights the lack of a “gold standard” anesthetic for infant inguinal hernia repair.

The issues and limitations of regional as well as GA in this special and often high‐risk group have prompted consideration of other anesthetic techniques. Many techniques have been described but have not permeated routine practice, presumably due to lack of applicability, or lack of uptake by surgeons, anesthetists or both.

The choice of anesthetic technique becomes a balance of minimizing the infant's perioperative anesthetic risk, providing optimal surgical conditions for the repair to occur, and the technical skill set of the entire treating team. Local and institutional factors may also play a role in the clinician's choice of anesthetic technique for an individual patient.

## 5 SPINAL BLOCK

The spinal block involves injection of local anesthetic into the subarachnoid space in an infant and has a duration of 45–85 min but has been reported up to 128 mins.^30^ It requires significant clinical skill of not only the anesthetist but the entire team including firm holding of the infant during insertion of the spinal needle.^30^ It is suitable predominantly for single sided surgery or simple bilateral hernias. Smaller younger infants are likely to fall asleep or remain settled without sedation, or with oral sucrose alone after administration of a spinal. Larger more robust infants can cry and move despite an adequate block and are more likely to result in block failure,^31^ leading some clinicians to put an upper weight, age or size limit on infants suitable for a spinal technique. Time pressure can add additional anxiety to the entire treating team to perform the surgery in a timely fashion.

The benefits of a spinal block predominantly relate to the significantly reduced respiratory complications when compared to GA. A meta‐analysis of studies specifically comparing spinal block to GA in infants born preterm found spinal block to have a lower rate of apneas (9% vs. 20% in the GA group), lower risk of post operative mechanical ventilation (1.9% vs. 13% in the GA group) and a lower rate of bradycardia. Overall, there was a 7.5% spinal failure rate, and surgical time was shorter in the spinal group.^32^

Spinal failure is generally defined as failure to find the subarachnoid space, insert local anesthetic or establish an adequate block. Failure rate is reported at 9–20%.^31^, ^33^ This failure rate may likely be higher in some settings such as clinicians or centres with a lower volume of practice. Surgery that continues for longer than the duration of the block can be managed in many ways, such as sedation or analgesic adjuncts, but can require conversion to GA. Conversion to GA during the surgery can provide increased stress to the treating team, and potential for increased risk to the patient.

The GA Compared to Spinal Anesthesia Study—Comparing Apnea and Neurodevelopmental Outcomes, a Randomized Controlled Trial (the GAS study) compared outcomes in infants undergoing either awake regional or GA and showed no significant difference in overall apnea rates, with only a small difference in favor of awake regional for early (less than 30 mins post op) apneas within the postanesthesia care unit.^34^ Importantly, this study was designed to look at neurodevelopment and consequently infants born less than 26 weeks gestational age and those with risk of adverse neurodevelopmental outcome were excluded from the study, which may have influenced this result.

## 6 CAUDAL BLOCK

Caudal block, injection of local anesthetic into the caudal epidural space, is one of the most commonly performed regional blocks in children for surgery^35^ either as a sole anesthetic technique or more often as an adjunct to sedation or GA. Successful block with a caudal in children has been reported as high as 94%.^36^

Caudal block has been shown to provide surgical anesthesia for up to 170 min and up to 15 h of residual analgesia.^37^ This provides anesthesia and analgesia for a significantly longer time than spinal block and has the additional benefit of providing post operative as well as intraoperative analgesia. The utilization of ultrasound guidance for caudal block placement has the advantage of further increasing its success rate.^38^ Its regular use in pediatric anesthesia and therefore increased clinician experience, high success rate and excellent safety profile make this a popular choice for infant inguinal hernia surgery.

Awake caudal block alone has been described for infant inguinal hernia surgery,^34^, ^39^ however it is regarded as technically difficult, unreliable and not considered a common anesthetic technique. One small randomized controlled trial showed improved success rate, postoperative analgesia and recovery time with caudal block alone compared to spinal block alone.^40^

Caudal and intravenous anesthesia without airway instrumentation is not a widespread technique but one that is gaining significant interest amongst the pediatric anesthesia community. A prospective case series of 228 infants undergoing surgery (not specific to inguinal hernia) with deep sedation (midazolam, nalbuphine and propofol) with caudal was effective for surgery with a low rate of post operative apnea (1%) and laryngospasm (2%), although 27% had sevoflurane for insertion of intravenous access.^41^

A randomized controlled trial comparing caudal and dexmedetomidine sedation in infants undergoing open inguinal hernia repair found a 90% success rate in completion of surgery without requirement for additional anesthetic agents. Compared to GA, the caudal dexmedetomidine group had a shorter duration of anesthesia, more bradycardia (none requiring intervention) but less hypotension and postoperative hypoxaemia.^42^ Similarly, a recent retrospective report of 20 infants undergoing laparoscopic inguinal hernia repair with caudal and intravenous anesthesia (remifentanil, dexmedetomidine and propofol) without airway instrumentation showed that this technique was an effective alternative to GA, with a low rate of postoperative desaturation and apneas.^43^ The same group have recently shown that the technique is a feasible alternative to GA for both open and laparoscopic hernia repair in infants.^44^

## 7 GENERAL ANESTHESIA

GA is the most common anesthetic technique performed on infants undergoing surgery in Europe.^1^ The benefits are optimal surgical conditions (an immobile patient, no time pressure) and a technique that is familiar to clinicians routinely involved in caring for infants. However, the downside is the common need for airway instrumentation, administration of opioids for analgesia and the potential concern of the effect of agents administered on the developing brain.

GA for infant inguinal hernia surgery has been shown to have significant risks of airway complications, early apneas and prolonged postoperative ventilation.^5^, ^6^, ^10^ A retrospective review of 485 infants undergoing hernia surgery with GA showed an overall respiratory complication rate of 9%, increasing to 35.5% in babies under 45 weeks post menstrual age at the time of surgery.^45^

A further review of 263 ex‐premature neonates undergoing inguinal hernia surgery showed an overall postoperative ventilator dependence of 8.3%, increasing to 14.7% in babies whose operation occurred prior to discharge.^46^ Post operative ventilator dependence of up to 25% has been reported.^47^

GA can be combined with a regional technique (spinal, caudal, epidural, ilioinguinal block or local anesthetic infiltration). This has the benefit of minimizing the amount of anesthetic agent used, and the need for opioids, which in term and preterm infants with the risk of opioid induced ventilatory impairment is an important consideration. A study in pediatric patients undergoing urology surgery have shown that the addition of a caudal to GA reduces postoperative pain and postoperative opioid requirements.^48^ A meta‐analysis of pediatric inguinal hernia repair comparing caudal to nerve block or wound infiltration found no significant difference in postoperative pain scores or rescue analgesia.^49^

## 8 AIRWAY MANAGEMENT FOR GENERAL ANESTHESIA

GA for inguinal hernia surgery can be performed with a variety of airway devices such as face mask ventilation, a laryngeal mask airway or an endotracheal tube. The risk of intraoperative and postoperative adverse airway events such as laryngospasm, bronchospasm and hypoxaemia has been consistently shown to be lower in infants with the use of a face mask or laryngeal mask airway compared to an endotracheal tube.^1^, ^12^, ^14^, ^50^ A randomized controlled trial of infants undergoing GA for a variety of surgeries showed a significant reduction in adverse events with the use of a laryngeal mask airway vs an endotracheal tube (18% vs 53%).^50^ Furthermore, laryngeal mask airways have been shown to be successfully used in infants undergoing inguinal hernia surgery.^51^ The lower risk of adverse events favors the use of face mask or laryngeal mask airway for infants undergoing inguinal hernia surgery, however other factors such as risk of reflux or regurgitation, requirement for high ventilation pressures, poor laryngeal mask airway fit and unfamiliarity with use of a laryngeal mask airway in small infants are considerations for use of an endotracheal tube.

High flow nasal oxygen insufflation has been used extensively in infants, particularly preterm, for many years. It is also an emerging mode of airway management in adult anesthesia and pediatric intensive care. It has been shown to be safe and effective in children undergoing anesthesia in several studies, including for airway surgery for children with difficult airways.^52^, ^53^ So far it does not appear to be used routinely for airway management in children undergoing inguinal hernia surgery, and its benefit is unclear in this setting.

## 9 NEURODEVELOPMENT

The effect of anesthesia on the developing brain is a topic of much debate, brought to light following findings of neuronal apoptosis and functional deficits in rodents exposed to general anesthetic medications in 2003.^54^ The U.S Food and Drug Administration in 2016 issued a Drug Safety Communication warning that “repeated or lengthy use of general anesthetic and sedation drugs in surgeries or procedures in children younger than 3 year old… may affect the development of children's brains”,^55^ a statement which was based largely on preclinical studies.

The only large randomized controlled trial aiming to assess neurodevelopmental effects of anesthesia published to date, the GA or Awake‐regional Anesthesia in infancy (GAS) study showed equivalence in full scale intelligence quotient between infants undergoing GA or regional anesthesia for inguinal hernia surgery.^56^ However, the results of the GAS study were included in two recent meta‐analyses; one which showed that childhood exposure to GA has a statistically significant increased risk of behavior problems and neurodevelopmental disorder diagnoses,^57^ and the other, that a brief or single early anesthetic exposure is not associated with objective measures of intelligence but is associated with a significant increase in parental reports of behavior problems.^58^ Evidence to date suggests the neurodevelopmental impact of anesthesia remains questionable and should encourage practitioners to continue avoiding or limiting general anesthetic exposure in infants where practicable.

## 10 CONSUMER ENGAGEMENT—PRIORITIES OF PARENTS

Consumer engagement is an emerging priority in the area of medicine and research. This has come about from the recognition that the question the clinical and research teams are asking, or the answers they are seeking, may not clearly align with the priorities of the consumer, in the case of infant hernia surgery the patient *and* their parents or immediate caregivers. Some groups have sought to identify the anesthesia research priorities of consumers and clinicians as a way of directing future research, with the Anesthesia Consumer Research Network (ACORN) showing that consumers have a top priority of safer anesthesia for children.^59^

There is little evidence in the literature on what parental perceptions are of the important priorities for anesthesia, and fewer still in infants undergoing hernia surgery. The only study relevant to infant inguinal hernia surgery was a survey of parents specifically exploring neurodevelopment, and found that 40% of parents had concerns about the impact of anesthesia on neurodevelopment, and more than 49% were moderately or very concerned about their child undergoing anesthesia.^60^ The authors could find no studies looking at parental insights of their infant undergoing anesthesia for infant hernia surgery, or what their concerns were.

## SUMMARY

As the most common operation performed in infants, inguinal hernia repair has been the topic of much debate and research over the past four decades. Despite this, there are still many questions that have not been definitively answered. To develop the ideal multi‐disciplinary perioperative care plan, the anesthetist must take into consideration the infant's specific comorbidities and risk, the surgical timing, technique and skill set, their own specific skills and preferences as well as the institutional setting. It may be helpful for clinicians to develop their own decision tree to select the optimal anesthetic technique for each infant. More work is needed to explore the parent/caregiver concerns and priorities regarding their infant's perioperative journey.

## REFLECTIVE QUESTIONS

What are the different techniques I can employ for infant inguinal hernia surgery and their relative pros and cons?

How is anesthetic or surgical management of an infant undergoing hernia surgery influenced by institutional factors and how can I modify these to improve my patient's perioperative management?

What is the impact of different surgical and anesthetic techniques in the resource limited perioperative healthcare system?

What are parental/caregiver perceptions of different anesthesia techniques and their priorities for infant inguinal hernia surgery?

## FUNDING INFORMATION

BSvUS is part funded by the Stan Perron Charitable Foundation and through a National Health and Medical Research Council Investigator Grant (2009322).

## CONFLICT OF INTEREST STATEMENT

Britta S von Ungern‐Sternberg is a section editor for Pediatric Anesthesia.

## Data Availability

Data sharing is not applicable to this article as no new data were created or analyzed in this study.
